# Evaluation of the Elastic Properties of Thirteen Silicone Interocclusal Recording Materials

**DOI:** 10.1155/2016/7456046

**Published:** 2016-09-26

**Authors:** Mieszko Wieckiewicz, Natalia Grychowska, Marek Zietek, Wlodzimierz Wieckiewicz

**Affiliations:** ^1^Division of Dental Materials, Faculty of Dentistry, Wroclaw Medical University, 26 Krakowska St., 50425 Wroclaw, Poland; ^2^Department of Prosthetic Dentistry, Faculty of Dentistry, Wroclaw Medical University, 26 Krakowska St., 50425 Wroclaw, Poland; ^3^Department of Periodontology, Faculty of Dentistry, Wroclaw Medical University, 26 Krakowska St., 50425 Wroclaw, Poland

## Abstract

*Background.* Addition silicones are popular as dental impression materials and are used in bite registration procedures.* Objective.* This study aimed to compare the postsetting elasticities and other mechanical properties of thirteen addition silicone interocclusal recording materials.* Materials and Methods.* The following materials were investigated: Colorbite D, Futar D, Genie Bite, Jet Blue Bite fast, Memoreg 2, O-Bite, Occlufast Rock, Omni-Bite Plus, Regidur i, Registrado X-tra, Regofix transparent, StoneBite, and Variotime Bite. Thirty specimens of each material were tested. The elasticities and strengths of the materials were measured with a universal testing machine, and computer software was used to determine the* E*-moduli, ultimate tensile strengths, and ultimate elongations of the specimens.* Results.* The results were subjected to statistical analysis using the Kruskal-Wallis test (*p* ≤ 0.05). The statistics revealed that the mean* E*-modulus values varied significantly across the materials (*p* = 0.000) and were highest for the StoneBite and Registrado X-tra and lowest for the Regofix transparent. The ultimate tensile strengths were highest for the Regofix transparent and Registrado X-tra (*p* = 0.000) and lowest for the Jet Blue Bite fast and Memoreg 2 (*p* = 0.000). The elongation percentages at the point of breaking varied significantly across the materials (*p* = 0.000); the lowest value was observed for the StoneBite, whereas the Regofix transparent nearly doubled original length.* Conclusions.* The authors concluded that materials with the high* E*-moduli and great ultimate tensile strengths may be most useful clinically. Registrado X-tra and StoneBite best met these criteria.

## 1. Introduction

The precise assessment of a patient's maxillomandibular relations is a key aspect of diagnosis and complex restorative therapy in oral rehabilitation. However, this assessment is insufficient without mounting the maxillary and mandibular casts in accordance with the recorded jaw relation on the articulator [1–8]. The interocclusal record is a registration of the positional relationship of the opposing teeth or arches and has become the most popular method of transferring maxillomandibular relations from the mouth to the articulator [2, 5]. An interocclusal record is mainly used to achieve the horizontal stability, which is essential to prevent the horizontal rotation or translation of the casts [9].

Materials used for occlusal registration include dental waxes, metal oxide pastes (such as zinc oxide pastes), acrylic resins, and elastomeric materials, such as polyethers and addition silicones [10]. The materials should possess attributes that are as similar as possible to the requirements for ideal bite registration material [3, 11]. These ideal requirements have been described as follows: (1) limited resistance before setting to avoid displacement of teeth or mandible during closure, (2) dimensional stability and resistance to compression after setting, (3) accurate recording of the incisal and occlusal surfaces of the teeth, (4) ease of handling, (5) biocompatibility with the tissues involved in the procedure, and (6) ease of verification [3, 11]. By knowing how well various registration materials meet these criteria, a dentist is more likely to choose the best material to obtain a precise and correct interocclusal record. Such records would allow the accurate placement of restorations, which would reduce the need for extensive adjustments and repeated clinical steps [2, 11].

Currently, polyether and polyvinyl siloxane bite recording materials are increasing in popularity due to their handling characteristics, accuracy, and dimensional stability [2, 6, 12, 13]. These materials are very similar to dental impression materials, but their properties following modifications via the addition of plasticizers and catalysts are unknown [2, 6, 7]. They do not require a carrier and are cost effective. Many papers have investigated the mechanical properties of addition silicone (A-silicone) bite recording materials including the dimensional stability, compression resistance, and accuracy [3, 13, 14]. However, some important characteristics, such as elasticity, remain unexamined. The major possible disadvantage of using elastomers is that any compressive force applied to these materials during the mounting of the casts may cause inaccuracies [5]. Compressive resistance depends, among other factors, on the thickness and stiffness of the material [2]. For these materials, it is important that a set material be both elastic enough to be easily removed from the mouth and rigid enough to resist deformation when forces are applied [10].

The purpose of this study was to evaluate the elasticities and other mechanical properties of thirteen A-silicone interocclusal recording materials. The null hypotheses were that the tested materials would not differ in* E*-modulus, ultimate tensile strength, and ultimate elongation percent.

## 2. Materials and Methods

Thirteen addition silicone interocclusal recording materials were studied: Colorbite D, Futar D, Genie Bite, Jet Blue Bite fast, Memoreg 2, O-Bite, Occlufast Rock, Omni-Bite Plus, Regidur i, Registrado X-tra, Regofix transparent, StoneBite, and Variotime Bite. This group of silicones was included to the study because of their popularity and worldwide availability. A detailed list of the studied materials is presented in Table 1.

Elasticity tests were performed on 390 specimens (30 of each material). The materials were supplied in the form of dental silicone cartridges. The cartridges had static mixing tips and were attached to a mixing gun. Each material was then injected into a silicone die (Dublosil 28, Emichem, Poland) that was coated with a thin layer of insulating agent (an aqueous solution of natural soap) for easy removal. Next, a rigid plastic plate was placed on the top of the silicone die with unset interocclusal registration material, and manual pressure was applied. The sample remained in the die for the setting time recommended by manufacturer. Thus, the prepared stripes measured 80 mm in length, 10 mm in width, and 2 mm in thickness. All 390 samples were obtained in a similar manner. The samples were stored in a room temperature in tightly sealed containers (~23°C) for 24 hours before testing [2, 8, 11].

A Z3 Nordic Transducer Teknik (Nordisk Transducer Teknik, Denmark) universal testing machine with a 3000 N Load Cell was applied for the tensile strength tests. The specimens were securely clamped using two grips. The distance between the grips was held constant at 50 mm. The two grips applied increasing tension to a specimen by stretching it in the vertical direction at a constant speed of 50 mm/min until the specimen broke [15]. The maximum load withstood by the specimen prior to breaking was recorded in N automatically by computer software (THSSD ZPM version: 1.0.1.57 R1). Next, the ultimate tensile strength (UTS) in MPa was calculated according to ASTM D412 specification [16, 17]:

(1) $$\begin{array}{c} UTS=\frac{F}{A}, \end{array}$$

where UTS is ultimate tensile strength of the stress at rupture, MPa. *F* is the force magnitude at rupture, MN. *A* is cross-sectional area of unstrained specimen, m^2^.

The* E*-modulus (used to describe the elasticity) and the ultimate elongation at break in millimeters were automatically determined by the software (THSSD ZPM version: 1.0.1.57 R1). The maximal elongation of specimen is expressed as the ultimate percentage elongation (% Eb) [15]. % Eb was determined according to the ASTM D412 specification [17]:

(2) $$\begin{array}{c} \%\;\;Eb=\frac{L-L_{o}}{L_{o}}\times 100, \end{array}$$

where % Eb is ultimate elongation in percent. *L* is observed distance between the grips at the point of specimen rupture. *L*_*o*_ is original distance between the grips (50 mm).

The data in the tables are presented as the mean values. The statistical analysis was performed using the Kruskal-Wallis test with the STATISTICA version 10 software (StatSoft Inc., Tulsa, OK, USA). The level of significance was set at *p* ≤ 0.05. Shapiro-Wilk tests revealed that the distributions of the values of some groups were not normal. Similarly, the variances between some groups were not homogeneous. The Kruskal-Wallis test was used for all analyses.

## 3. Results

The tests of statistical significance rejected the null hypotheses. The tested materials differed in* E*-modulus, ultimate tensile strength, and ultimate elongation.

### 3.1. *E*-Modulus Results

The analysis indicated statistically significant differences in the* E*-modulus values between the tested materials.  Table 2 demonstrates the results of the analysis for all thirteen materials (*H* = 327.42;  *p* = 0.000). The most elastic material was the Regofix transparent (*E*-modulus is 6.53 MPa). The StoneBite and Registrado X-tra exhibited the highest* E*-moduli of all the tested materials (167.19 MPa and 107.38 MPa, resp.).

To better understand the statistical relationships between the most rigid materials, the authors performed an additional statistical test that included only the first four materials listed in Table 2, that is, StoneBite, Registrado X-tra, O-Bite, and Variotime Bite (*H* = 89.68;  *p* = 0.000). The additional Kruskal-Wallis test revealed that the* E*-modulus of the StoneBite was significantly greater than that of the Registrado X-tra (*p* = 0.000). The statistical significance of the difference in the* E*-moduli of the Registrado X-tra and O-Bite was weak (*p* = 0.045). There was no significant difference between the O-Bite and Variotime Bite (*p* = 0.222).

The Genie Bite, Occlufast Rock, Regidur i, and Omni-Bite Plus had similar elastic properties according to an analysis of all thirteen tested materials. To better understand the statistical relationships between only the four above-mentioned silicones, an additional statistical test was performed. The additional Kruskal-Wallis test revealed that the Genie Bite had the highest* E*-modulus value (*H* = 13.47;  *p* = 0.004).

### 3.2. Ultimate Tensile Strength

The comparisons revealed significant differences in the ultimate tensile strengths of the studied silicones.  Table 3 presents the results of the analysis of all thirteen materials (*H* = 217.73;  *p* = 0.000). The statistics divided the materials into 5 subgroups with no significant differences in the UTSs of the silicones in the subgroups (*p* > 0.05). The 1st subgroup included Regofix transparent and Registrado X-tra, which exhibited the highest UTSs of all of the tested recording media (5.67 MPa and 5.53 MPa, resp.). The 2nd subgroup contained Omni-Bite Plus, Colorbite D, and StoneBite which showed high UTSs (4.67 MPa, 4.43 MPa, and 4.37 MPa, resp.). Moderate loads were sustained in the 3rd subgroup, which included Occlufast Rock, O-Bite, Variotime Bite, and Genie Bite (4.18 MPa, 4.08 MPa, 4.07 MPa, and 3.92 MPa, resp.). The 4th subgroup included Regidur i and Futar D and exhibited low tensile strengths (3.47 MPa and 3.45 MPa, resp.). The lowest UTSs was observed in the 5th subgroup, which included Jet Blue Bite fast and Memoreg 2 (2.95 MPa and 2.67 MPa, resp.).

### 3.3. Ultimate Elongation

Statistically significant differences were also observed in the elongation at break values (% Eb).  Table 4 provides detailed characteristics of the mean values and the results of the analysis of all thirteen materials (*H* = 355.56;  *p* = 0.000). Nearly every material was significantly different from the others. The Regofix transparent yielded the highest % Eb (95.97%), whereas the StoneBite exhibited the lowest elongation of only 3.69%.

**Figure 1:**
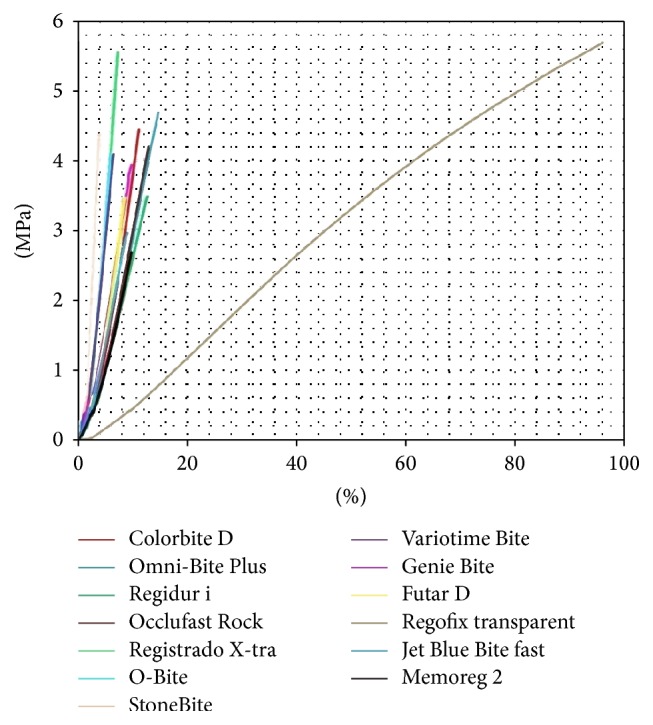
The stress-strain curves, showing the relations between achieved mean values of ultimate tensile strengths [MPa] and ultimate elongations [%] for the tested materials.

The stress-strain curves, showing the relations between UTSs and ultimate elongations for the tested materials, are presented in Figure 1.

## 4. Discussion

Due to introduction of different interocclusal recording materials, dentists encounter difficulties in the selection of the optimum material for the registration and transfer of occlusal records to the articulator [11]. Manufacturers compete against each other for getting consumers by promoting the advantages of their products; thus, clinicians need reliable sources for product description.

The advanced management of occlusion requires proper examination, recording, storage, and transferring the relation of dental arches to the articulator [18]. Moreover, inaccuracy in the transfer of information between dentist and technician regarding occlusal contacts can cause problems when fabricating indirect restorations, which can result in frustration for the dentist, technician, and patient [19]. The occlusal instability caused by incorrect final restoration may cause inappropriate function of the stomatognathic system [20, 21].

Since 1756, when the first interocclusal record was made, many materials have been used for maxillomandibular registration including dental waxes, acrylic resin, zinc oxide-eugenol pastes, and elastomers [10, 11]. Elastomeric materials are growing in popularity in the prosthodontics [18]. They can be applied in situations when a dentist needs to accurately reproduce the intraoral conditions. Traditionally, they are used as dental impression materials; however, due to their properties, they are also applicative interocclusal recording media. Megremis et al. [10] in ADA Professional Product Review of eight addition silicones investigated several of their characteristics. The results showed that, after removing the deforming force, all of the them recovered between 98 and 100% of their original shape. That indicates their ability to recover elastically after removal from the mouth. All of the silicone bite registration materials were able to reproduce the 20-micrometer-wide line completely over the entire length of the detail reproduction test block. All of the evaluated silicones exhibited a linear dimensional change of 0.5 percent or less across 14 days, even after undergoing disinfection. Chun et al. [22] examined polymerization shrinkage strain of interocclusal recording materials. The lowest setting shrinkage strain showed O-Bite (polyvinylsiloxane-based material): 0.18 ± 0.03–0.16 ± 0.03% at 5, 7, and 10 min, followed by polyether-based material, whereas dimethacrylate-based material had the highest degree of shrinkage. In the study of Anup et al., dimensional change and accuracy of polyvinyl siloxane bite registration material were also statistically significant but clinically insignificant [6]. Campos and Nathanson [5] examined the compressibility of two addition silicones interocclusal record materials, analyzing the changes of maxillomandibular relations at the condyle region. There was no significant change in maxillomandibular relations when forces up to 1 kg were applied to stabilize the casts.

Previous studies have proven that wax and zinc oxide-eugenol are not reliable as interocclusal registration materials due to substantial linear changes that occur even within the first hour [5, 14]. The vertical changes that occur with waxes (aluminum wax, 11 ± 3 microm; hydrocarbon wax compound, 12 ± 3 microm) are greater than those of elastomers (addition silicones, from 0 ± 1 microm to 2 ± 1 microm; polyether, −2 ± 2 microm) with loading forces up to 1 kg [23]. The main disadvantages of wax relative to elastomers are the flow characteristics caused by fluctuation in temperature, with zinc oxide-eugenol paste being the material with lower resistance to compression when compared to elastomers [3, 24]. Moreover, waxes do not accurately reproduce the incisal and occlusal forms of teeth, spread laterally in closure, and cause patient to close into undesirable patterns [6]. The advantages of wax are low cost and ease of manipulation [9]. Although polyether has been found to be a more dimensionally stable interocclusal recording material than polyvinyl siloxane, both can be used to relate working casts during mounting procedures without significant vertical displacements [11, 25].

The accuracy of an interocclusal record is influenced not only by the material properties but also by the recording technique [6]. Based on the existing intraoral conditions, the clinician needs to decide the most suitable material-technique combination. According to Prasad et al., when good intercuspation exists between the teeth no record may be needed, whereas if there is poor intercuspation, a full arch or segmental record may be made using elastomeric materials or a segmental record may be made only over the prepared tooth/teeth using rigid materials like wax, plaster, resin, or paste [9]. Nowadays, instead of using a physical occlusal registration material, the CAD-CAM-generated dental casts can be mounted by using a best-fit alignment algorithm without any physical interocclusal record. To mount the physical dental casts made by CAD-CAM technology, the buccal surfaces of the maxillary and mandibular teeth are scanned in maximum intercuspation and then the scanning data are analyzed with computer software [26]. According to Solaberrieta et al., virtual occlusion is a valid procedure for the location of the mandibular cast. The contacts observed in the virtual environment were significantly more accurate than those of the physical ones and provided more objective and meaningful data [27]. Moreover, digital analysis of occlusion provides additional information on occlusal contact pattern, including the quantification of force, sequence of contact, and occlusal-disocclusal timing [21].

In the study, authors investigated the* E*-modulus, ultimate tensile strength, and ultimate elongation. The elastic modulus is defined as the change in stress with an applied strain and is inversely proportional to the elasticity of the material. The higher the* E*-modulus, the lower the elasticity. Meththananda et al. [28] confirmed that hardness of elastomeric dental materials is related to* E*-modulus; that is, higher* E*-modulus was associated with greater hardness of the material. The hardness of material is related in a general way to its compressive strength [29] and is defined as the relative resistance that a surface of the material imposes against the penetration of a harder body. Ultimate tensile strength describes the ability of interocclusal materials to resist tearing forces appearing during removal from the mouth when the material goes into undercuts or potential damage during transport or laboratory procedures. Ultimate elongation is maximal extension of the material up to its breaking point. It is associated with above-mentioned physical properties. The ideal material should be elastic enough to be easily removed from the mouth and rigid enough to resist deformation when forces are applied during articulation. It should be durable and resistant to damage during transport and shortage.

Tejo et al. [11] suggested that limited elasticity can cause adverse effect such as the possibilities of breaking during removal from the mouth, increased initial resistance, and difficulty in verification due to brittleness. However, excessive elasticity can influence the bite record or cause undesirable shifts during adjustments of the positions of the casts in the articulator. Cracked or fractured interocclusal material may not allow casts to be mounted so that an articulator does not accurately reproduce the relationships of the mandible to the skull in the temporomandibular joints.

According to Nagrath et al. [2], the ability of an interocclusal recording material to resist compressive force is critical. Compressive force is commonly exerted on the interocclusal recording material during articulation and may cause inaccuracy and distortion of the final restoration [2, 30]; thus, minimal pressure should be applied to articulated casts during mounting when using elastomeric interocclusal recording materials [2]. The deformation may vary with the thickness and the stiffness of the recording material [2, 31, 32]. The record should be minimally thick, and an optimal material should exhibit minimal distortion during compression; therefore, the material should have substantial dimensional stability [2, 33].

Parker et al. [16] reported that, in aqueous environments, all polymeric materials will absorb water and elastic moduli can affect the level of water uptake. In the study, authors compared two experimental silicones, which did not differ in the UTS and percentage elongation. However, the lower* E*-modulus of one silicone resulted in a lower restraining force and thus greater water uptake in comparison to another studied silicone [16]. The addition of a hydrophilic agents to silicone-based materials can compromise the materials' mechanical integrities, especially those with low moduli [16]. Cadenaro et al. [34] indicated inverse correlations between the* E*-moduli of some resin blends and their Hoy solubility parameters; that is, higher elasticity was associated with greater hydrophilicity. Unfortunately, literature does not concern water uptake of additive silicones for interocclusal registration; however, as they are polymeric materials, the elasticities may have considerable influence on water absorption.

Due to mentioned characteristics, this independent research has a strong clinical aspect because the potential recipients will be able to consider which of the tested silicones is close to have an optimal elasticity for clinical use. It has to be emphasized that stiffness of silicone interocclusal recording material is one of the most important mechanical features in a practical point of view because of the jaw relation reproduction ability and accuracy [18].

Regofix transparent exhibited the greatest UTS and the greatest break resistance. However, this material was the most elastic material among those tested; therefore, the authors do not recommend Regofix transparent as the material of choice for bite registration. Due to limited stiffness, some inaccuracies during mounting casts may occur.

The* E*-modulus seems to be inversely proportional to the degree of elongation. The StoneBite, Registrado X-tra, O-Bite, Variotime Bite, and Futar D, that is, the five materials with the greatest* E*-moduli, exhibited the lowest elongations at break. StoneBite, which exhibited by far the highest stiffness among the tested materials, ruptured after 3.69% elongation, which was the lowest value observed in the ultimate tensile strength test. During the test, only the velocity of grip was constant. The force applied to the samples per second was variable across materials. For example, the materials of the 5th subgroup, which withstood the smallest loads, exhibited moderate ultimate elongation at break values. As mentioned previously, Jet Blue Bite fast and Memoreg 2 exhibited the lowest UTSs and quite high elasticities. Their* E*-moduli were greater than only that of Regofix transparent. The clinical application of these materials may also increase the risk of errors.

Regidur i and Futar D composed another 4th subgroup with lower UTSs than most of the tested materials. Their UTSs were greater than only those of the 5th subgroup. Regidur i was more elastic than Futar D. The materials with limited strengths and small to moderate elasticities should be applied cautiously due to the risk of cracks and fractures that may occur during removal from the mouth or the trimming away of the excess.

Registrado X-tra and the materials of the 2nd subgroup, including Omni-Bite Plus, Colorbite D, and StoneBite, exhibited satisfactory UTSs. Registrado X-tra was the second-most durable and rigid material; therefore, Registrado X-tra was found to be the optimal material. Although StoneBite was the most rigid material, it withstood a high load. The risk of cracks and fractures due to its limited elasticity appears to be overcome with very good strength; thus, StoneBite may also be recommended. Omni-Bite Plus and Colorbite D exhibited very good break resistance, but Colorbite D, in contrast to elastic Omni-Bite Plus, had moderate* E*-modulus value. The other materials displayed intermediate values for the studied characteristics.

The authors proved that the tested A-silicone bite recording materials have different elasticities and mechanical properties. As laboratory conditions do not always predict clinical behavior, further studies should be performed to evaluate whether these differences have perceptible clinical implications in the registration of the centric relation.

## 5. Conclusions

Previous research has indicated that high* E*-modulus and great ultimate tensile strength may be appropriate for judging the clinical usefulness of bite registration material. Registrado X-tra and StoneBite best met these criteria. Nonetheless, the other materials that exhibited moderate values for the* E*-moduli and UTSs can be successfully used, that is, O-Bite, Occlufast Rock, Variotime Bite, and Genie Bite.

## Figures and Tables

**Table 1:** Interocclusal recording materials included in the study.

Material	Lot number	Manufacturer
Colorbite D	148137	Zhermack, BadiaPolesine, Italy
Futar D	112151	Kettenbach GmbH & Co. KG., Eschenburg, Germany
Genie Bite	110826	Sultan Healthcare, York, PA, USA
Jet Blue Bite fast	C30559	Coltène/Whaledent AG, Altstätten, Switzerland
Memoreg 2	380036	Heraeus Kulzer GmbH, Hanau, Germany
O-Bite	683563	DMG Chemisch-Pharmazeutische Fabrik GmbH, Hamburg, Germany
Occlufast Rock	132241	Zhermack, BadiaPolesine, Italy
Omni-Bite Plus	20242	Omnident-Dental-Handels GmbH, Rodgau, Germany
Regidur i	203743/2912	Bielefelder Dentalsilicone GmbH & Co. KG, Bielefeld, Germany
Registrado X-tra	1229190	VOCO GmbH, Cuxhaven, Germany
Regofix transparent	207001.09	Dreve Dentamid GmbH, Unna, Germany
StoneBite	208132.10	Dreve Dentamid GmbH, Unna, Germany
Variotime Bite	380076	Heraeus Kulzer GmbH, Hanau, Germany

**Table 2:** Comparison of *E*-modulus mean values for the tested materials.

Material	*E*-modulus [MPa] Mean value ± SD
(1) StoneBite	167.19 ± 19.59^(1)^
(2) Registrado X-tra	107.38 ± 11.36^(1)^
(3) O-Bite	93.94 ± 13.32^(2)^
(4) Variotime Bite	83.59 ± 8.76^(3)^
(5) Futar D	64.85 ± 9.05^(4)^
(6) Colorbite D	57.38 ± 7.52^(5)^
(7) Genie Bite	55.47 ± 7.27^(6)^
(8) Occlufast Rock	48.41 ± 7.38^(6)^
(9) Regidur i	48.11 ± 10.47^(6)^
(10) Omni-Bite Plus	47.88 ± 12.07^(6)^
(11) Jet Blue Bite fast	43.34 ± 9.66^(7)^
(12) Memoreg 2	42.22 ± 6.87^(7)^
(13) Regofix transparent	6.53 ± 2.34^(8)^

^(1)^ *p* < 0.05 in comparison to (5)–(13).

^(2)^ *p* < 0.05 in comparison to (6)–(13).

^(3)^ *p* < 0.05 in comparison to (7)–(13).

^(4)^ *p* < 0.05 in comparison to (1), (2) and (11)–(13).

^(5)^ *p* < 0.05 in comparison to (1)–(3) and (13).

^(6)^ *p* < 0.05 in comparison to (1)–(4) and (13).

^(7)^ *p* < 0.05 in comparison to (1)–(5).

^(8)^ *p* < 0.05 in comparison to (1)–(10).

**Table 3:** Comparison of ultimate tensile strength mean values for the tested materials.

Material	Ultimate tensile strength [MPa] Mean value ± SD
(1) Regofix transparent	5.67 ± 1.06^(1)^
(2) Registrado X-tra	5.53 ± 1.13^(1)^

(3) Omni-Bite Plus	4.67 ± 0.87^(2)^
(4) Colorbite D	4.43 ± 0.83^(2)^
(5) StoneBite	4.37 ± 0.62^(2)^

(6) Occlufast Rock	4.18 ± 0.63^(3)^
(7) O-Bite	4.08 ± 0.80^(3)^
(8) Variotime Bite	4.07 ± 0.69^(3)^
(9) Genie Bite	3.92 ± 0.52^(3)^

(10) Regidur i	3.47 ± 0.51^(4)^
(11) Futar D	3.45 ± 0.60^(4)^

(12) Jet Blue Bite fast	2.95 ± 0.57^(5)^
(13) Memoreg 2	2.67 ± 0.47^(5)^

^(1)^ *p* < 0.05 in comparison to (6)–(13).

^(2)^ *p* < 0.05 in comparison to (10)–(13).

^(3)^ *p* < 0.05 in comparison to (1), (2) and (12), (13).

^(4)^ *p* < 0.05 in comparison to (1)–(5).

^(5)^ *p* < 0.05 in comparison to (1)–(9).

**Table 4:** Comparison of percentage ultimate elongation mean values for the tested materials.

Material	Ultimate elongation [%] Mean value ± SD

(1) Regofix transparent	95.97 ± 19.42^(1)^
(2) Omni-Bite Plus	14.58 ± 1.34^(2)^
(3) Occlufast Rock	12.78 ± 1.40^(3)^
(4) Regidur i	12.56 ± 1.12^(3)^
(5) Colorbite D	11.01 ± 1.28^(4)^
(6) Memoreg 2	9.66 ± 1.25^(5)^
(7) Genie Bite	9.65 ± 1.27^(5)^
(8) Jet Blue Bite fast	8.93 ± 1.15^(6)^
(9) Futar D	8.43 ± 1.08^(7)^
(10) Registrado X-tra	7.12 ± 1.01^(8)^
(11) Variotime Bite	6.36 ± 0.64^(9)^
(12) O-Bite	5.81 ± 0.61^(10)^
(13) StoneBite	3.69 ± 0.53^(11)^

^(1)^ *p* < 0.05 in comparison to (5)–(13).

^(2)^ *p* < 0.05 in comparison to (6)–(13).

^(3)^ *p* < 0.05 in comparison to (8)–(13).

^(4)^ *p* < 0.05 in comparison to (1) and (10)–(13).

^(5)^ *p* < 0.05 in comparison to (1)-(2) and (11)–(13).

^(6)^ *p* < 0.05 in comparison to (1)–(4) and (12)-(13).

^(7)^ *p* < 0.05 in comparison to (1)–(4) and (13).

^(8)^ *p* < 0.05 in comparison to (1)–(5).

^(9)^ *p* < 0.05 in comparison to (1)–(7).

^(10)^ *p* < 0.05 in comparison to (1)–(8).

^(11)^ *p* < 0.05 in comparison to (1)–(9).

## Abbreviations

UTS: Ultimate tensile strength
% Eb: Ultimate elongation in percent
ASTM: American Society for Testing and Materials
A-silicone: Addition silicone
ADA: American Dental Association.

## Additional Points

*Limitations of the Study.* Main limitation was that in vitro study results may differ compared to in vivo use of tested material and its behavior. Moreover, there was the difficulty in preparing a dumbbell-shaped specimen, which was recommended in ASTM D412 specification [17]. Nonetheless, in accordance with the ASTM D412, specimens may also be in shape of straight pieces; however, straight specimens tend to break in the grips of universal testing machine.
